# Evaluation of the Informational Content and Readability of US Lung Cancer Screening Program Websites

**DOI:** 10.1001/jamanetworkopen.2019.20431

**Published:** 2020-01-31

**Authors:** Staci M. Gagne, Florian J. Fintelmann, Efren J. Flores, Shaunagh McDermott, Dexter P. Mendoza, Milena Petranovic, Melissa C. Price, Justin T. Stowell, Brent P. Little

**Affiliations:** 1 Department of Radiology, Brigham and Women’s Hospital, Boston, Massachusetts; 2 Thoracic Imaging and Intervention Division, Department of Radiology, Massachusetts General Hospital, Boston; 3 Department of Radiology, Mayo Clinic, Jacksonville, Florida

## Abstract

**Importance:**

The internet is an important source of medical information for many patients and may have a key role in the education of patients about lung cancer screening (LCS). Although most LCS programs in the United States have informational websites, the accuracy, completeness, and readability of these websites have not previously been studied.

**Objective:**

To evaluate the informational content and readability of US LCS program websites.

**Design, Setting, and Participants:**

This cross-sectional study assessed US LCS program websites identified on September 15, 2018. A standardized checklist was used to assess key informational content of each website, and text was analyzed for reading level, word count, and reading time. Links to US websites of national advocacy organizations with LCS program content were tabulated. All functional LCS program websites in Google internet search engine results using the search terms *lung cancer screening*, *low-dose CT screening*, and *lung screening* were included in the analysis.

**Main Outcomes and Measures:**

Radiologists used a standardized checklist to evaluate content, and readability was assessed with validated scales. Website word count, reading time, and number of links to outside LCS informational websites were assessed.

**Results:**

A total of 257 LCS websites were included in the analysis. The word count ranged from 73 to 4410 (median, 571; interquartile range, 328-909). The reading time ranged from 0.3 to 19.6 minutes (median, 2.5; interquartile range, 1.5-4.0). The median reading level of all websites was grade 10 (interquartile range, 9-11). Only 26% (n = 66) of websites had at least 1 web link to a national website with additional information on LCS. There was wide variability regarding reported eligibility age criteria, with ages 55 to 77 years most frequently cited (42% [n = 108]). Only 56% (n = 143) of websites mentioned smoking cessation. The subject of patient cost was mentioned on 75% (n = 192) of websites. Although major LCS benefits, such as detection of lung cancer, were discussed by most (93% [n = 239]) websites, less than half of the websites (45% [n = 115]) made any mention of possible risks associated with screening.

**Conclusions and Relevance:**

There appears to be marked variability in the informational content of US LCS program websites, and the reading level of most websites is above that recommended by the American Medical Association and the National Institutes of Health. Efforts to improve website content and readability may be warranted.

## Key Points

**Question:**

Is the informational content of lung cancer screening program websites in the United States accurate, complete, and presented at an appropriate reading level for most patients?

**Findings:**

In this cross-sectional study of 257 lung cancer screening program websites, there was large variability regarding information on enrollment criteria, benefits and risks, logistics, and costs of lung cancer screening. The reading level of the websites was consistently above that of the average adult in the United States and the level recommended by the American Medical Association and the National Institutes of Health.

**Meaning:**

Efforts to improve the content and readability of program websites may be warranted to improve patient understanding of lung cancer screening.

## Teaser

This cross-sectional study evaluates the informational content and readability of US lung cancer screening program websites.

## Introduction

In the United States, lung cancer has the highest cancer-specific mortality rate. The National Lung Screening Trial (NLST) showed a 20% reduction of lung cancer–specific mortality with low-dose computed tomography (LDCT) after 3 annual screenings compared with a chest radiography control arm.^1^ After publication of the NLST results, the US Preventive Services Task Force and the Centers for Medicare & Medicaid Services (CMS) soon followed with recommendations for LDCT lung cancer screening (LCS).^2^ This led health care plans offered under the Affordable Care Act’s Health Insurance Marketplace, as well as most private plans, to cover certain preventive care services, such as LDCT LCS, without patient cost sharing.^3^ Despite the proven benefits of LCS among eligible patients, the insurance coverage, and the increasing number of LCS facilities across the United States, the rate of LCS participation remains low. It is estimated that only 1.9% of up to 8 million screening-eligible US adults have been screened.^4^

Accessible and comprehensive patient information on eligibility, benefits, risks, costs, and logistics of LCS may be important for facilitating understanding of this still underused service. Patients eligible for LCS may have questions and may experience confusion and anxiety about the screening process.^5,6,7^ Lung cancer screening may prompt follow-up diagnostic examinations, procedures, visits, and therapies that are often subject to patient cost sharing in the form of deductibles and copayments, in addition to the potential costs of travel, missed work, and similar expenditures.^8^ Prior evidence has shown that medical information for patients is typically written at a reading level higher than the grade 8 level of the average US adult.^9^ Many current and prior smokers may have cognitive and health literacy challenges, underscoring the need for accurate information written at an accessible level.^10,11^

Shared decision-making, in which clinicians discuss eligibility, benefits, and risks of LCS with patients as mandated by the CMS, is intended to address questions and concerns, but many health care professionals have found it difficult to cover LCS optimally within the time constraints of a patient visit.^12,13,14,15^ Many patients turn to the internet for more information. Most adults in the United States have used the internet to gather health information,^16^ and internet-based resources have been shown to increase LCS participation among patients.^17^ Numerous national advocacy organizations have created websites with LCS information, but the websites of LCS programs may be the first or primary source of information for many patients. The objective of this study was to evaluate the informational content and readability of US LCS program websites.

## Methods

### Website Selection

In this cross-sectional study, all functional LCS program websites were identified with a Google internet search using the search terms *lung cancer screening*, *low-dose CT screening*, and *lung screening* on September 15, 2018. We used a Mozilla Firefox browser in incognito mode connected to a virtual private network server to prevent any customization to the search algorithm that may result from location, cookies, or user account information.^18^ Websites that were duplicates or that did not have functional links were excluded. The protocol for this study was exempt from review by the Massachusetts General Hospital Institutional Review Board because it used only publicly available information. Quality in the reporting of our findings was assessed using the Strengthening the Reporting of Observational Studies in Epidemiology (STROBE) reporting guidelines for cross-sectional studies.^19^

### Content Analysis

Included websites were randomly assigned for analysis to 1 of 9 thoracic radiologists (all authors) participating in an LCS program. Radiologists reviewed text, images, videos, and other file attachments on the main landing pages of LCS programs and linked pages with a standardized checklist that evaluated the presence of information related to LCS.^2,20,21^ Readability was assessed with validated scales. Website word count, reading time, and number of links to outside LCS informational websites were assessed. The content subjects included LCS eligibility criteria, monetary costs and insurance coverage, benefits, risks, and logistics of LCS. All links to national websites providing lung cancer and/or lung screening information (eg, shouldiscreen.com, lungcanceralliance.org [now go2foundation.org], and cancer.org) were counted. External links to the LCS program’s institution were not included in the content analysis. The lead and senior authors (S.M.G. and B.P.L., respectively) confirmed the accuracy of entries after the primary content assessment; any discrepancies were resolved by consensus between the 2, and any missing data were added after additional website review.

### Classification of Practice Type and US Geographic Region

The practice setting of each LCS program website was classified as academic, community, hybrid, or other. The academic classification was used when the institution was a teaching hospital affiliated with a medical school. Community practice was used for community hospitals not associated with an academic institution. Hybrid status was assigned when the institution was a health network or system with private, community, and sometimes academic components. Programs classified as other did not fit any of these categories and included private radiology practices not primarily affiliated with an institution. The US geographic region was determined by zip codes.

### Readability Analysis

Text was manually copied into Microsoft Word (Microsoft Corp) and cleaned of formatting and of content, including figures, captions, hyperlinks, advertisements, and addresses. Readability was assessed with a commercial web-based suite of tools (readable.com). The average grade-level score comprised the mean of 5 widely used and validated readability indexes.^22,23,24,25,26^ The Flesch-Kincaid score is based on the average number of words per sentence and the mean number of syllables per word.^22^ The Gunning-Fog index tabulates reading level with a formula using the number of sentences, the number of words, and the number of words with 3 syllables or more.^23^ The Coleman-Liau formula requires the number of characters and sentences.^24^ The Simple Measure of Gobbledygook (SMOG) selects 10 sentences from the beginning, middle, and end of a text and uses the number of words with 3 syllables or more.^25^ The Automated Readability Index represents difficulty through a ratio of the number of letters in a word to the number of words in a sentence.^26^

### Statistical Analysis

Microsoft Excel (version 16.27), including the XLSTAT add-in (version 2019.1.1; Addinsoft), was used for data processing and statistics. Descriptive statistics were used to characterize the informational content and readability of each LCS program website. The practice type of the associated institution, the US geographic region, and the presence of material regarding LCS eligibility criteria, benefits, risks, costs, and logistics were summarized. The medians and SDs of website readability scores, word count, and reading time were calculated. Scatterplots and boxplots were generated to show variability in website readability scores and word counts.

## Results

In total, 257 unique US LCS program websites of 269 results returned by the Google internet search were included in the analysis. The other 12 search results were links to nonexistent web pages, duplicates, or websites without LCS-related information. The practice types and US geographic regions of these LCS programs are listed in Table 1.

**Table 1.** Characteristics of US Lung Cancer Screening Program Websites

Characteristic	No. (%) (N = 257)
Practice type	
Academic	48 (19)
Community	61 (24)
Hybrid	97 (38)
Other	51 (20)
US geographic region	
Midwest	60 (23)
Northeast	69 (27)
South	91 (35)
West	37 (14)

### Enrollment Criteria

Information about having a smoking history of 30 or more pack-years was listed on 241 of 257 websites (94%), and data on being an active smoker or having quit within 15 years was detailed on 231 websites (90%) (Table 2). There was wide variability regarding reported eligibility in age criteria, with ages 55 to 74, 55 to 77, and 55 to 80 years cited on 14% (n = 35), 42% (n = 108), and 16% (n = 42) of websites, respectively; 19% (n = 48) of websites mentioned more than 1 age range. Lack of signs or symptoms of cancer was mentioned on 120 websites (47%), and willingness and/or ability to undergo curative treatment was mentioned by only 18 websites (7%).

**Table 2.** Informational Content of US Lung Cancer Screening Program Websites

Website Content	Websites Containing Content, No. (%) (N = 257)
**Lung Cancer Screening Enrollment Criteria**	
Smoking history	
History of smoking ≥30 pack-years	241 (94)
Active smoker or having quit within 15 y	231 (90)
Age requirement, y	
55-74	35 (14)
55-77	108 (42)
55-80	42 (16)
Multiple	48 (19)
Other	18 (7)
Lack of signs or symptoms of cancer	120 (47)
Willingness and/or ability to undergo curative treatment	18 (7)
**Logistics of Screening**	
Smoking cessation mentioned	143 (56)
Website link or phone number for smoking cessation	94 (37)
Frequency of LDCT examinations mentioned	108 (42)
Annual	87 (34)
Annual follow-up duration of 3 y	9 (4)
Annual until quit smoking ≥15 y or exceed age criterion	8 (3)
Follow-up not otherwise specified	2 (1)
Other	2 (1)
Meeting with navigator or other screening personnel	64 (25)
Possible lung nodule or lung cancer screening clinic referral	60 (23)
Possible need for biopsy or further testing	56 (22)
**Costs**	
Any mention of patient cost	192 (75)
Medicare or private insurance coverage	131 (51)
Not free, amount mentioned	31 (12)
Not free, no amount given	21 (8)
Free	9 (4)
**Benefits**	
Low radiation dose of LDCT	239 (93)
Early detection of lung cancer	238 (93)
Mortality reduction	210 (82)
Ease of LDCT	164 (64)
Magnitude of mortality reduction benefit mentioned	157 (61)
Correctly referenced a 20% reduction of lung cancer mortality	115 (45)
Unspecified reduction	41 (16)
Incorrect % reduction	1 (0.4)
Potential for curative treatment of early-stage lung cancer	38 (15)
**Risks**	
Any mention of possible risks associated with screening	115 (45)
Further imaging or procedures	102 (40)
Exposure to radiation	98 (38)
False-positive results	94 (37)
False-negative results	51 (20)
Anxiety or worry	50 (20)
Overdiagnosis	32 (13)
Procedural complications	26 (10)
Out-of-pocket costs	17 (7)

Abbreviations: LDCT, low-dose computed tomography.

### Screening Logistics

Frequency of LDCT examinations was mentioned on 108 of 257 websites (42%) and was correctly described as annual on only 87 websites (34%) (Table 2). Nine websites (4%) incorrectly listed an annual follow-up duration of 3 years. The possibility to stop annual follow-up once smoking cessation had been achieved for 15 or more years or whether a patient had aged out was mentioned on 8 websites (3%). Smoking cessation was mentioned on only 143 websites (56%), and a telephone number or a link to cessation resources was provided on only 94 websites (37%). Additional details on the informational content of all 257 LCS program websites are listed in Table 2.

### Costs of Screening

The subject of patient cost was mentioned on 192 of 257 websites (75%) (Table 2). Medicare or private insurance was listed as a mode of coverage by 131 websites (51%). A specific dollar amount was provided by only 31 websites (12%), whereas cost without a specific amount was mentioned on only 21 websites (8%). Nine websites (4%) indicated that LCS was free.

### Benefits and Risks of Screening

In terms of major LCS benefits, the low radiation dose of LDCT was mentioned on 239 of 257 websites (93%), and the ease of LDCT was mentioned on 164 websites (64%) (Table 2). Early detection of lung cancer was mentioned on 238 websites (93%). A mortality reduction with LCS was mentioned on 210 websites (82%). Although 115 websites (45%) correctly referenced a 20% reduction of lung cancer mortality, an unspecified reduction was mentioned on 41 websites (16%), and 1 website (0.4%) described an incorrect reduction of 15% to 20%. The potential for curative treatment of early-stage lung cancer was mentioned on only 38 websites (15%).

Possible risks related to screening were mentioned on less than half of 257 websites (115 [45%]) (Table 2). Mentioned risks included further imaging tests or invasive procedures (102 [40%]), exposure to radiation (98 [38%]), false-positive results (94 [37%]), false-negative results (51 [20%]), anxiety or worry (50 [20%]), overdiagnosis (32 [13%]), procedural complications (26 [10%]), and out-of-pocket costs (17 [7%]).

### Links to National Websites With Information on Lung Cancer and/or LCS

Only 66 of 257 LCS program websites (26%) had at least 1 web link to a national website with additional information on LCS. Most commonly, these web links were cancer.gov (22 [9%]), shouldiscreen.com (15 [6%]), and lungcanceralliance.org (now go2foundation.org) (14 [5%]) (Table 3).

**Table 3.** Links to National Websites in US Lung Cancer Screening Program Websites

Website Domain Address	Organization	Links to National Websites, No. (%) (N = 257)
cancer.gov	National Cancer Institute	22 (9)
shouldiscreen.com	University of Michigan	15 (6)
lungcanceralliance.org (now go2foundation.org)	GO_2_ Foundation for Lung Cancer	14 (5)
cancer.org	American Cancer Society	11 (4)
lung.org	American Lung Association	10 (4)
acr.org	American College of Radiology	8 (3)
nccn.org	National Comprehensive Cancer Network	8 (3)
radiologyinfo.org	Radiological Society of North America	7 (3)
uspreventiveservicestaskforce.org	US Preventive Services Task Force	5 (2)
pubmed.gov	US National Library of Medicine article abstract links	4 (2)
cms.gov	Centers for Medicare & Medicaid Services	4 (2)
cdc.gov	Centers for Disease Control and Prevention	3 (1)
thoracic.org	American Thoracic Society	2 (1)

### Readability, Word Count, and Reading Time

The median reading level of all websites was grade 10 (interquartile range [IQR], 9-11) (Figure, A). The minimum reading level was grade 5, and the maximum reading level was grade 16. Only 4 of 257 websites had reading levels at or below the grade 6 level recommended by the American Medical Association for patient materials.^27^ An additional 41 websites were written at or below the grade 7 to grade 8 level recommended by the National Institutes of Health, for a total of 45 websites meeting this less stringent guideline.^9^ The word count ranged from 73 to 4410 (median, 571; IQR, 328-909) (Figure, B). The reading time in minutes ranged from 0.3 to 19.6 (median, 2.5; IQR, 1.5-4.0). A description of differences across US geographic regions is summarized in Table 4.

**Figure.**
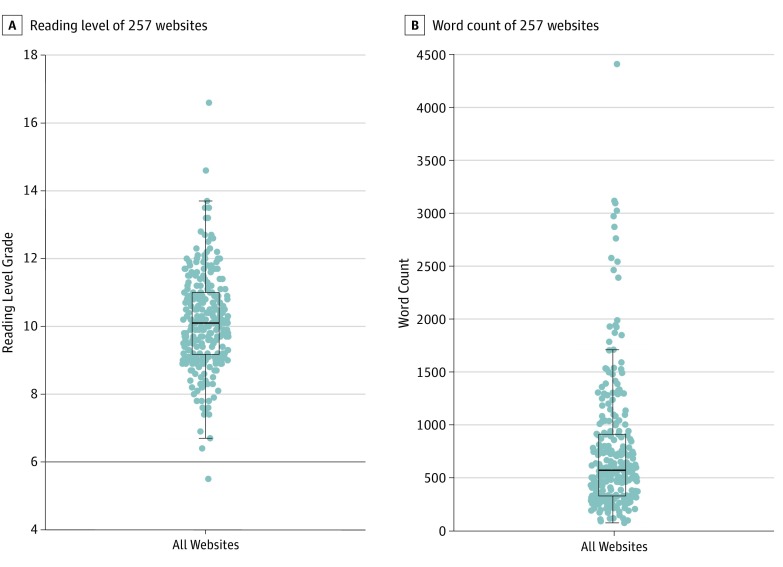
Reading Level and Word Count of 257 US Lung Cancer Screening Program Websites Reading levels were assessed as the mean of 5 validated readability scores (Flesch-Kincaid score,^22^ Gunning-Fog index,^23^ Coleman-Liau formula,^24^ SMOG [Simple Measure of Gobbledygook],^25^ and Automated Readability Index^26^). A, The horizontal line indicates the grade 6 reading level recommended by the American Medical Association for medical literature for patients.^27^ B, Word counts were tabulated for the relevant portions of each website. Each dot indicates a website. The middle line indicates the median; the lower box end, the lower quartile; the upper box end, the upper quartile; the lower whisker, minimum excluding outliers; and the upper whisker, maximum excluding outliers.

**Table 4.** Readability, Word Count, and Reading Time of 257 US Lung Cancer Screening Program Websites

Text Attribute	Median (IQR) [Range]
**Reading Level Grade**	
All	10.1 (9.2-11.0) [5.5-16.6]
Northeast	10.2 (9.6-11.1) [5.5-14.6]
South	9.9 (9.2-11.1) [6.4-16.6]
Midwest	10.0 (9.0-10.8) [6.9-13.2]
West	10.1 (9.0-10.8) [6.7-13.5]
**Word Count, No.**	
All	571 (328-909) [73-4410]
Northeast	666 (380-1082) [171-4410]
South	476 (322-768) [106-2762]
Midwest	632 (367-1018) [89-3024]
West	563 (454-870) [73-1988]
**Reading Time, min**	
All	2.5 (1.5-4.0) [0.3-19.6]
Northeast	3.0 (1.7-4.8) [0.8-19.6]
South	2.1 (1.4-3.4) [0.5-12.3]
Midwest	2.8 (1.6-4.5) [0.4-13.4]
West	2.5 (2.0-3.9) [0.3-8.8]

Abbreviation: IQR, interquartile range.

## Discussion

Because most US adults use the internet to find health information,^16^ the accuracy of health care websites is of paramount importance. Of those who search online for information on LCS, 77% begin with a search engine^16^ and likely find the websites evaluated in this study. In our comprehensive analysis of the LCS program websites, we found inconsistencies, incompleteness, and a reading level above that of the average US adult (grade 8 level).^9^ Unlike other studies^11,28^ investigating the reading level of online LCS information, the present study evaluated the content of LCS program websites, which may serve as a primary source of information for many LCS-eligible persons. The LCS program websites offer an opportunity for comprehensive coverage of enrollment criteria, logistics, costs, benefits, and risks of screening. Such information can supplement the shared decision-making process that occurs during a patient visit, which is often constrained by time and by competing health concerns. Websites can also link to national organization websites that have peer-reviewed decision-making tools.

### Enrollment Criteria and Screening Logistics

Prior investigations have found low rates of familiarity with LCS eligibility criteria among primary care clinicians. In a recent study,^29^ only 31% of clinicians knew the correct eligibility criteria for LCS. Our data demonstrated that LCS program websites provide conflicting information that may confuse patients and their health care clinicians. For example, the upper age limit for LCS was sometimes listed as age 80 years, as recommended by the US Preventive Services Task Force^21^; age 77 years, the upper age limit covered by CMS^2^; and age 74 years, which was the upper age limit in the NLST and is also listed on websites by the American Cancer Society,^30^ the American Society of Clinical Oncology,^31^ and the American Lung Association.^32^ If present, description of follow-up protocols included both annual LDCT without an end point and annual LDCT for 3 years. These content discrepancies can be confusing and potentially misleading.

The CMS requires that smoking cessation interventions be made available to patients before screening at imaging facilities, but only 56% (143 of 257) of websites mentioned smoking cessation, and even fewer (37% [94 of 257]) offered a telephone number or a link to smoking cessation resources. Park et al^33^ demonstrated that LCS may provide an excellent opportunity for intervention for current smokers because older smokers are more likely to use the help given and stop smoking. In addition, Cataldo^34^ found that high-risk smokers are interested in quitting; therefore, the information in the setting of LCS should be made available to them at every opportunity.

### Costs of Screening

In a survey of 338 LCS-eligible persons in 2015, a total of 78.4% (265 of 338) indicated interest in information about the cost of LCS.^34^ As part of the Affordable Care Act, LCS is covered by the CMS and private insurance plans, but additional costs as a result of screening, such as travel cost, wage loss, and follow-up procedures, can also encumber and possibly dissuade LCS-eligible persons.^35^ Although cost of LCS was mentioned on 75% (192 of 257) of websites, only 51% (131 of 257) mentioned coverage by Medicare or private insurance. Only 7% (17 of 257) of websites described out-of-pocket expenses. Patients understandably have questions about the immediate and downstream costs of LCS, and lack of such information may discourage participation in such programs.^36^

### Benefits and Risks of Screening

The low radiation dose of LCS computed tomography (CT) (93% [239 of 257]) and the early detection of lung cancer (93% [238 of 257]) were the most commonly mentioned benefits of screening on the websites we studied. The fear of radiation exposure is a known barrier to LCS participation,^34,36^ similar to breast cancer screening^37^; therefore, the screening benefits are essential information to provide.

Possible risks of LCS were addressed by 45% (115 of 257) of the websites, which may represent a missed opportunity for patient education. In a study by Kanodra et al,^38^ clinicians often encountered patients who knew that CT was the initial step in LCS but did not ask about follow-up testing. In another study,^39^ many health care clinicians also reported substantial obstacles to comprehensive discussion of LCS with patients, such as insufficient clinical time, inadequate staff, and competing patient clinical concerns. An investigation by Brenner et al^14^ identified the challenges of shared decision-making and reported that LCS was discussed for only 59 seconds (range, 16-219 seconds) on average. Not surprisingly, the studied conversations were poor in observed quality and failed to meet basic skill criteria per the Observing Patient Involvement in Decision Making (OPTION) scale for reviewing benefits and risks of LCS. Comprehensive and accurate LCS program website information may facilitate a more efficient and informed shared decision-making process for patients and clinicians.

### Links to National Websites With Information on LCS

The low incidence (26% [66 of 257]) of links on LCS program websites to national informational websites is surprising, especially given the lack of comprehensive information on many local websites. Professionally created, peer-reviewed LCS informational websites are provided by renowned organizations, such as the American Cancer Society,^40^ the American Lung Association,^41^ and the Lung Cancer Alliance.^42^ In addition, widely acclaimed decision aids are also available online; examples include shouldiscreen.com,^43^ the Agency for Healthcare Research and Quality,^44^ the Veteran Health Administration,^45^ and the American Thoracic Society.^46^ Among participants who used shouldiscreen.com, Lau et al^47^ showed that there was an increase in LCS knowledge and a decrease in decisional conflict. However, without links to national informational websites, LCS-eligible individuals may not take advantage of these resources.

### Readability, Word Count, and Reading Time

The accessibility of health information has been an ongoing concern for the medical community, with many patient materials written at a higher reading level than that of the average American adult. Although the American Medical Association recommends a grade 5 or grade 6 reading level for patient medical information,^27^ only 4 of 257 LCS program websites met this requirement. Forty-five of 257 websites met the grade 7 or grade 8 level recommended by the National Institutes of Health.^9^ Most US adults read at a grade 8 or grade 9 reading level, and 20% read at a grade 5 level or lower, but health care materials are often written at a grade 10 level or higher.^48^ Of the websites we studied, 138 of 257 (54%) were written at a grade 10 level and higher.

Several prior studies^9,11,18,28^ have assessed the readability of health care information for patients, a handful of which evaluated LCS patient information available on the internet.^11,28^ However, these studies were limited to a smaller subset of patient educational materials rather than LCS program websites and did not evaluate information content. The most commonly used website readability scores are the Automated Readability Index^26^ (which was designed for health education materials), the Coleman-Liau Index,^24^ the SMOG,^25^ the Gunning-Fog index,^23^ and the Flesch-Kincaid score^22^ (which is found in the Microsoft Word grammar checker^9^). Similar to our findings, Hansberry et al^28^ found a mean (SD) grade 12.6 (2.7) reading level among 80 online LCS patient education articles. Haas et al^11^ reviewed 46 LCS informational websites and calculated a mean (SD) reading level of grade 10.6 (2.2). Although readability is an important consideration for assessing accessibility of website text, word count and reading time may alter overall effectiveness by influencing reader engagement and retention. In our study, word count ranged from 73 to 4410, and reading time ranged from 0.3 to 19.6 minutes. We are not aware of formal recommendations for optimal length of online health information, but eye tracking studies of websites from a variety of industries by the Nielsen Norman Group^49^ have shown that users rarely read beyond the third screenful of information and would rather scroll than click through a series of short web pages. Our study provides information on the total reading time of LCS program websites, but future observational studies examining the optimal content, length, and structure of LCS program websites may be warranted.

### Limitations

This study has limitations. Readability tests provide an estimate of the understandability of text; however, they do not account for the content conveyed by videos, pictures, and schematics that may be included on websites. Content review can be subject to omissions and interpretive errors, but we attempted to minimize these by using a structured template for scoring websites, by randomly distributing websites to subspecialists with experience in LCS, and by reviewing results for accuracy and completeness. Although creating a comprehensive content checklist is challenging, we attempted to score the most substantial topics as highlighted in the recent LCS literature. Finally, although our study may not have included every US LCS program website, we used websites found in the results of the most widely used search engine.

## Conclusions

This study found that information provided on the websites of LCS programs varies widely. Reading levels frequently exceed those recommended by the American Medical Association and the National Institutes of Health. Minimizing inconsistencies, including appropriate content, and presenting information at an accessible reading level may facilitate dissemination of LCS information and potentially increase LCS participation.
